# Toll-like receptor 3 modulates the behavioral effects of cocaine in mice

**DOI:** 10.1186/s12974-018-1130-8

**Published:** 2018-03-23

**Authors:** Ruiming Zhu, Qian Bu, Dengqi Fu, Xue Shao, Linhong Jiang, Wei Guo, Bo Chen, Bin Liu, Zhengtao Hu, Jingwei Tian, Yinglan Zhao, Xiaobo Cen

**Affiliations:** 1 National Chengdu Center for Safety Evaluation of Drugs, State Key Lab of Biotherapy/Collaborative Innovation Center of Biotherapy, West China Hospital, West China Medical School, Sichuan University, #28 Gaopeng Street, High Technological Development Zone, Chengdu, 610041 China; 2 Healthy Food Evaluation Research Center, Department of Food Science and Technology, College of Light Industry, Textile and Food Engineering, Sichuan University, Chengdu, 610065 China; 3 School of Pharmacy, Key Laboratory of Molecular Pharmacology and Drug Evaluation (Yantai University), Ministry of Education, Collaborative Innovation Center of Advanced Drug Delivery System and Biotech Drugs in Universities of Shandong, Yantai University, Yantai, 264005 China

**Keywords:** Cocaine, TLR3, Drug addiction, NF-κB

## Abstract

**Background:**

The nucleus accumbens in the midbrain dopamine limbic system plays a key role in cocaine addiction. Toll-like receptors (TLRs) are important pattern-recognition receptors (PPRs) in the innate immune system that are also involved in drug dependence; however, the detailed mechanism is largely unknown.

**Methods:**

The present study was designed to investigate the potential role of TLR3 in cocaine addiction. Cocaine-induced conditioned place preference (CPP), locomotor activity, and self-administration were used to determine the effects of TLR3 in the rewarding properties of cocaine. Lentivirus-mediated re-expression of *Tlr3* (LV-TLR3) was applied to determine if restoration of TLR3 expression in the NAc is sufficient to restore the cocaine effect in TLR3^−/−^ mice. The protein levels of phospho-NF-κB p65, IKKβ, and p-IκBα both in the cytoplasm and nucleus of cocaine-induced CPP mice were detected by Western blot.

**Results:**

We showed that both TLR3 deficiency and intra-NAc injection of TLR3 inhibitors significantly attenuated cocaine-induced CPP, locomotor activity, and self-administration in mice. Importantly, the TLR3^−/−^ mice that received intra-NAc injection of LV-TLR3 displayed significant increases in cocaine-induced CPP and locomotor activity. Finally, we found that TLR3 inhibitor reverted cocaine-induced upregulation of phospho-NF-κB p65, IKKβ, and p-IκBα.

**Conclusions:**

Taken together, our results describe that TLR3 modulates cocaine-induced behaviors and provide further evidence supporting a role for central pro-inflammatory immune signaling in drug reward. We propose that TLR3 blockade could be a novel approach to treat cocaine addiction.

**Electronic supplementary material:**

The online version of this article (10.1186/s12974-018-1130-8) contains supplementary material, which is available to authorized users.

## Background

Cocaine is one of the most widely abused drugs and poses serious social, medical, and economical problems [1]. Repeated use of cocaine causes long-lasting changes in the brain’s reward circuitry, a crucial component of which is the nucleus accumbens (NAc) [2]. Cocaine triggers cellular and molecular alterations that lead to stable changes in neuroplasticity in the NAc [3, 4]. Animal behavioral studies have demonstrated that proinflammatory cytokines of the central immune system are involved in cocaine-induced pathological alterations in the brain [5]. Traditionally, these changes have been considered to be the results of cocaine-induced neurotoxicity [6, 7]. However, more recent studies have shown that cocaine-induced activation of central immune signaling contributes substantially to the behavioral effects of cocaine [8–10].

Toll-like receptors (TLRs) are evolutionarily conserved pattern-recognition receptors (PRRs) that are critically involved in host defense mechanisms in many species, including plants and humans [11]. Animal studies have demonstrated the participation of the innate immune system, especially the TLR family, in the behavioral response to multiple drugs of abuse [12–14]. Blockade of TLR4 suppresses opioid-induced conditioned place preference (CPP) and reduces opioid self-administration in mice [15]. Moreover, blockade of TLR4 also suppresses cocaine-induced extracellular dopamine in the NAc as well as cocaine CPP and self-administration [9]. Opioid activation of TLR4 contributes to drug reinforcement [15], and mice lacking TLR4 are largely protected against ethanol-induced behavioral associated effects during alcohol abstinence [16].

Among the TLRs, TLR3 recognizes small interfering RNAs, viral double-stranded RNA, and self-RNAs derived from damaged cells [17]. Unlike other TLRs, which are Myd88-dependent, TLR3 initiates a TIR-domain-containing adapter-inducing interferon-β (TRIF)-dependent signaling pathway that leads to the activation of NF-κB for the induction of inflammatory cytokine genes [18, 19]. Previous studies have reported that the expression of ncRNAs, particularly lncRNAs, is affected by cocaine [20, 21]. These ncRNAs can directly activate the TLR3 signaling pathway through TLR3 [22, 23]. However, there is no evidence linking TLR3 to cocaine-induced behaviors.

NF-κB is a critical transcriptional factor that regulates the transcription of a large number of genes, including those involved in immune and inflammatory response, cell death, and proliferation [24]. Studies have shown that NF-κB plays an important role in the cocaine rewarding effect [13, 25]. NF-κB is activated by cocaine and plays an important role in synaptic plasticity and memory [26]. Under normal conditions, the NF-κB subunits are downstream of TLR3 and are confined to the cytoplasm by the inhibitory protein IκB. Studies have confirmed TLR3 can recruit TRIF and promote an alternative pathway that leads to NF-κB activation for induction of proinflammatory cytokines, such as IL6, IL10, and IFN-β. NF-κB is mainly localized in the cytoplasm with an inactive form bound to an inhibitory κB protein (IκBα). In response to stimulation, the IKK complex phosphorylates IκBα, which undergoes proteasome degradation, allowing NF-κB to translocate to the nucleus to induce proinflammatory gene expression.

In the present study, we aimed to define the role of TLR3 in cocaine addiction and its associated inflammatory immune signaling. We hypothesized that TLR3 signaling may be involved in cocaine behavioral effects. We found that both TLR3 deficiency and intra-NAc injection of TLR3 inhibitor significantly attenuated cocaine-induced CPP, locomotor activity, and self-administration. Our findings show that TLR3, a component of the innate immune system, plays a role in cocaine-induced behavior.

## Methods

### Animals

C57BL/6 background TLR3^−/−^ knockout (KO) mice were purchased from Jackson Laboratories (#009675, Bar Harbor, ME). Male and female homozygous mice were bred to generate TLR3^−/−^ homozygotes, and the male TLR3^−/−^ mice were selected for the experiments. Male C57BL/6 wild-type (WT) mice were purchased from Vital River (Beijing, China). All of the animals were housed four per cage in temperature (23 ± 3 °C) and light (12:12 light to dark, lights on from 7:00 A.M. to 7:00 P.M.)-controlled rooms with standard rodent chow and water available ad libitum. The animals were acclimatized for 7 days before experiments and habituated to handling for at least 3 days before behavioral testing.

### Reagents and antibodies

The test substance cocaine-HCl was purchased from the National Institute for the Control of Pharmaceutical and Biological Products (#171210, Beijing, China). Cocaine was dissolved in 0.9% saline. Antibody for phospho-NF-κB subunit p65 was purchased from Abcam (Cambridge, UK). Antibodies for phospho-IκBα (Ser32/36), IKKβ, and β-actin were purchased from Cell Signaling Technology (Boston, USA). The Nuclear and Cytoplasmic Protein Extraction Kit and BCA Protein Assay Kit were purchased from Beyotime Institute of Biotechnology (Jiangsu, China). All of the other reagents were of analytical grade.

### Conditioned place preference (CPP) procedure

**Fig. 1:**
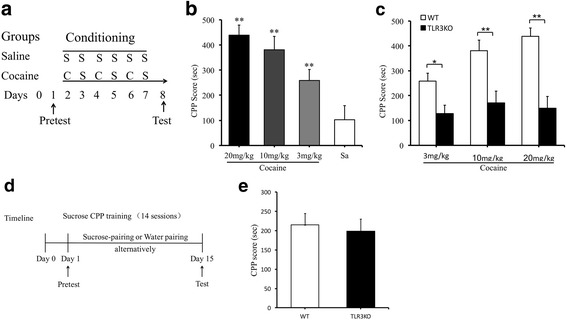
TLR3 deficiency attenuates cocaine CPP, not sucrose CPP. **a** Experimental schedule for the cocaine-induced CPP tasks, cocaine injection (C), saline injection (S). **b** Cocaine produces significant CPP. **p < 0.01, *n* = 10/saline group, *n* = 13/cocaine groups (3, 10, 20 mg/kg). **c** TLR3 deficiency attenuates cocaine-induced CPP. *p < 0.05, **p < 0.01, *n* = 13/WT groups (3, 10, 20 mg/kg cocaine), *n* = 12/TLR3 KO groups (3, 10, 20 mg/kg cocaine). **d** Experimental schedule for the sucrose-induced CPP tasks. **e** Sucrose-induced CPP acquisition has no significant differences between TLR3KO and WT mice. *n* = 12/WT groups, *n* = 12/TLR3 KO groups. Data are presented as the mean ± SEM

The CPP test in mice was performed as previously described with slight modifications [27]. Briefly, the CPP test was conducted by using a shuttle box which consisted of three chambers: two large conditioning chambers and a small neutral start chamber. One of the large conditioning chamber had black walls, while the other had white walls. The start chamber’s walls were gray. The mice were acclimatized to the apparatus for three consecutive days for 15 min each day before the pre-conditioning phase (day 1, pre-test). During the pre-conditioning phase, mice were placed in the neutral chamber and allowed to explore all compartments freely for 15 min. The time spent in each chamber was recorded, and unbiased mice were randomly assigned to two groups—the control group and the cocaine group. After injection of cocaine in the cocaine group (20 mg/kg, *i.p.*) and injection of saline in the control group (0.9% sodium chloride) on days 2, 4, and 6, mice were confined to the corresponding conditioning compartment by closing the removable wall for a period of 15 min. After injection of saline in both the cocaine group and saline group on days 3, 5, and 7, mice were confined to the opposite conditioning chamber for the same amount of time. In the post-conditioning phase (day 8), mice were placed in the neutral chamber with free access to both compartments for 15 min, and the time spent in each compartment was measured (Fig. 1a). The results were expressed as the time spent in the cocaine-paired chamber minus the time spent in the saline-paired chamber during CPP testing.

The procedure for training sucrose CPP was modified from previous studies [28, 29]. Before the pretest and the training session, both WT and TLR3^−/−^ mice were subjected to water restriction for 2 h. During the training phase for the CPP test, unbiased mice were placed in the central area and allowed to explore both chambers freely for 15 min on the pretest day (day 1). The mice were then confined to the 15% sucrose solution or water-paired compartment by closing the removable wall for 30 min for 14 alternating days. On the post-test day (day 15), the mice were placed in the central area with free access to both compartments for 15 min with no sucrose or water identical to the pretest and the time spent in each compartment was measured (Fig. 1d). The results were expressed as the time spent in the sucrose solution-paired chamber minus the time spent in the water-paired chamber during CPP testing.

### Cocaine self-administration

Mice were anesthetized before a sterilized catheter was surgically inserted into the right jugular vein [30]. Mice were singly housed with chow and water available ad libitum for a week. The training paradigm consisted of mice having 2-h daily access to cocaine during which they were given access on a fixed ratio one (FR1) schedule to a cocaine-paired nose poke, which initiated an intravenous injection of cocaine (0.75 mg/kg/infusion) upon response. After each response/infusion, the poke was retracted and a stimulus light was illuminated for a 20-s timeout period.

### Locomotor activity

The locomotor activity measurement was performed using four activity chambers (square white acrylic cage, 48 cm in length) that were equipped with a top unit that included a camera. The mice were individually placed into cages, which were situated in a sound-attenuated room. Locomotor activity was defined as the number of interruptions of a beam during the observation period. Automated tracking was performed using EthoVision 7.0 software (EthoVision 7.0; Noldus Information Technology, Leesburg, VA). Locomotor activity sessions were conducted once daily. Each mouse was placed in a locomotor activity chamber followed by cocaine (20 mg/kg, *i.p.*) or saline administration, and the locomotor activity was measured for 15 min as previously described with slight modifications [31].

### Construction of lentiviral vectors for expression of TLR3

Viral plasmids EX-Mm13690-Lv201 (pEZ-CMV-Tlr3-SV40-eGFP-IRES-puro) and EX-NEG-Lv201 were purchased from GeneCopoeia for the production of lentivirus expressing TLR3 (LV-TLR3) and lentivirus expressing GFP (LV-GFP). The open reading frame sequence of LV-TLR3 is listed in Additional file 1. These two vectors contained the enhanced green fluorescence protein (GFP) coding sequence, which allowed for the identification of the infected cells. All vector insertions were confirmed by dideoxy-sequencing. Recombinant lentivirus was produced by transient transfection in HEK293T cells using EX-Mm13690-Lv201 (to yield LV-TLR3) and EX-NEG-Lv201 (to yield LV-GFP). Viral titers were determined by infection of 293T cells and GFP visualization (1.35 × 10^8^ TU/ml). Aliquots were kept at − 80 °C.

### Stereotaxic injection of LV-TLR3 in the NAc

For stereotaxic surgery, mice were anesthetized with ketamine/xylazine and installed in a small-animal stereotaxic instrument, and the cranial surface was exposed. Using a precision Hamilton micro-syringe, mice were bilaterally infused with 0.5 μl of virus into the NAc at a 10° angle (AP + 1.6; ML ± 1.5; DV − 4.4) at a rate of 0.1 μl/min. After surgery, animals were allowed to recover for 1 week.

### Tissue isolation

At the end of each CPP test, the mice were sacrificed by rapid decapitation. The NAc was removed from the brain, snap-frozen in liquid nitrogen, and stored at − 80 °C until protein extracts were prepared.

### Western blot analysis

For whole protein extraction, NAc tissues were homogenized and then dissolved with protein extraction agent which was supplemented with phenylmethanesulfonyl fluoride (PMSF, Sigma-Aldrich, St Louis, MO, USA) and EDTA-free protease inhibitor cocktail tablets (Roche, Bielefeld, Germany). The protein concentration of the supernatants was determined by the Bradford assay kit (Bio-Rad, California, USA). The extraction and isolation of nuclear and cytoplasmic proteins were performed according to the Nuclear and Cytoplasmic Protein Extraction Kit instructions (#P0028, Beyotime, Jiangsu, China) as described previously [32]. The protein extracts from the NAc were separated using 6–12% SDS-PAGE gels and then transferred to PVDF membrane (Bio-Rad). After blocking with 3% BSA for 1 h, the membranes were incubated with the primary antibodies at 4 °C overnight. After washing three times with TBST, the blots were incubated with secondary antibodies conjugated to horseradish peroxidase (ZSGB-BIO, Beijing, China) for 1 h at room temperature. Immunoreactivity was visualized using ECL Western blotting detection reagents and then analyzed through scanning densitometry. The blots were visualized by enhanced chemiluminescence using Kodak X-OMAT BT film (Carestream Health, Inc., USA).

### Statistical analysis

Statistical significance was measured using an unpaired two-tailed Student’s test for IKK-β and p-IκBα protein analysis when two groups were being compared using SPSS Statistics 21 software. One-way analysis of variance (ANOVA) followed by Bonferroni post hoc tests was used to determine significance for cocaine-induced CPP and protein analysis when more than two groups were being compared using SPSS Statistics 21 software. For locomotor activity, as well as comparisons of nuclear and cytoplasmic proteins, two-way ANOVA followed by Bonferroni post hoc tests were performed using GraphPad Prism 5 software because these experiments contained multiple groups. All values included in the figure legends represent mean ± SEM (*p < 0.05; **p < 0.01; ***p < 0.001).

## Results

### TLR3 deficiency attenuates cocaine CPP

An unbiased CPP procedure was used to determine whether TLR3 deficiency affected the rewarding properties of cocaine. The timeline of the procedure was depicted in Fig. 1a. During habituation to the CPP apparatus, no side preference was present in any of the groups. Cocaine induced a dose-dependent (3, 10, and 20 mg/kg, i.p.) increase in time spent in the drug-paired chamber in WT mice (Fig. 1b; *F*_(3,45)_ = 13.299, **p < 0.01). However, compared with WT mice, the preference time of the TLR3 knockout mice induced by various doses cocaine were suppressed significantly (Fig. 1c; *F*_(5,69)_ = 13.184, **p < 0.01). We used sucrose CPP to determine if TLR3^−/−^ mice have more reward learning deficits that impair their ability to perform these behaviors. Both TLR3^−/−^ and WT mice showed a preference for the environment associated with sucrose solution, but no significant group differences were observed (Fig. 1e; *t*_(22)_ = 1.302, *p >* 0.05). These results suggest that genetic blockade of TLR3 impairs cocaine-CPP.

### TLR3^−/−^ mice exhibit weakened cocaine-induced locomotor activity

**Fig. 2:**
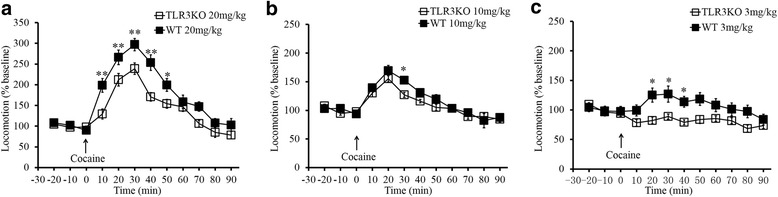
TLR3-/- mice exhibit significantly reduced locomotor activity. Locomotor activity was assessed in 10-min epochs over the 90-min recording session in WT and TLR3 deficiency mice in 20 mg/kg (**a**), 10 mg/kg (**b**), 3 mg/kg (**c**) cocaine. *p <0.05, **p <0.01, *n*=12/WT groups, *n*=12/TLR3 KO groups. Data are presented as the mean ± SEM

Before cocaine administration, the basal locomotor activity of mice during three consecutive 10-min intervals were recorded and used as a covariate in the analysis of cocaine-induced locomotor activity. Locomotor activity was recorded during nine consecutive 10-min intervals after cocaine injection, and the total distance traveled during each interval was calculated. The locomotor activity of the mice increased promptly during the first three consecutive intervals after cocaine treatment. The time course of the distance traveled by the mice compared to baseline is shown in Fig. 2. We found that TLR3^**−/−**^ mice displayed decreased locomotor activity in response to each dose of cocaine as compared to WT mice (Fig. 2; *F*_(5,66)_ = 11.000, *p < 0.05.). In the 20 mg/kg cocaine trials, TLR3^**−/−**^ mice exhibited a greater decrease in hyperlocomotor activity (Fig. 2a; *F*_(1,22)_ = 4.214, **p < 0.01.). These results suggest that cocaine-induced hyperlocomotor activity can be weakened by TLR3 deficiency.

### TLR3 deficiency decreases cocaine self-administration

**Fig. 3:**
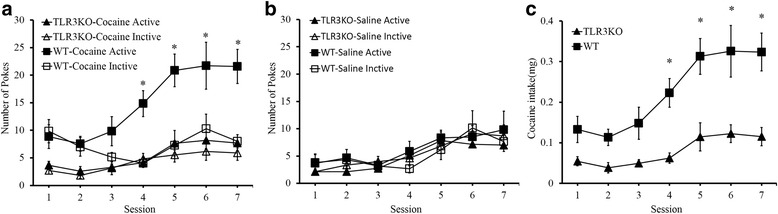
TLR3 deficiency decreases cocaine self-administration. **a** WT mice exhibit stronger cocaine-seeking behavior than TLR3-knockout mice after 4 sessions. *p < 0.05, *n* = 7/WT group, *n* = 7/TLR3KO group. **b** TLR3 knockout mice have no significant difference in saline self-administration compared to WT mice, *n* = 7/WT group, *n* = 7/TLR3KO group. **c** WT mice show significantly different cocaine intake compared to TLR3 deficient mice after 4 sessions, *p < 0.05, *n* = 7/WT group, *n* = 7/TLR3 KO group. Data are presented as the mean ± SEM

We next assessed whether TLR3 knockout could affect cocaine self-administration. Acquisition of cocaine self-administration and performance on a number of nose pokes for TLR3^**−/−**^ mice was compared to that for WT mice. Cocaine treatments produced a clear discrimination in WT mice for the active poke over the inactive poke throughout acquisition and stable self-administration (Fig. 3a; *F*_(3,30)_ = 16.194, *p < 0.05). This result indicates that the WT mice exhibited strong cocaine-seeking behavior. Moreover, we found a remarkable decrease in the number of active pokes in the TLR3^−/−^ mice compared to that in the WT group. In contrast, the number of active pokes and inactive pokes between WT and TLR3^−/−^ mice both had no difference during saline treatments (Fig. 3b; *F*_(3,24)_ = 0.380, *p >* 0.05). In addition, the cocaine intake of all mice increased over time, and there was a clear discrimination between WT mice and TLR3^**−/−**^ mice (Fig. 3c; *F*_(1,15)_ = 21.559, *p < 0.05). Collectively, WT mice exhibited stronger cocaine-seeking behaviors than the TLR3^−/−^ mice, and TLR3 deficiency reduced such behaviors. These results are similar to the aforementioned findings in the locomotor activity and CPP tests.

### Restoring TLR3 expression in the NAc reverts deficits in cocaine-induced behavior

**Fig. 4:**
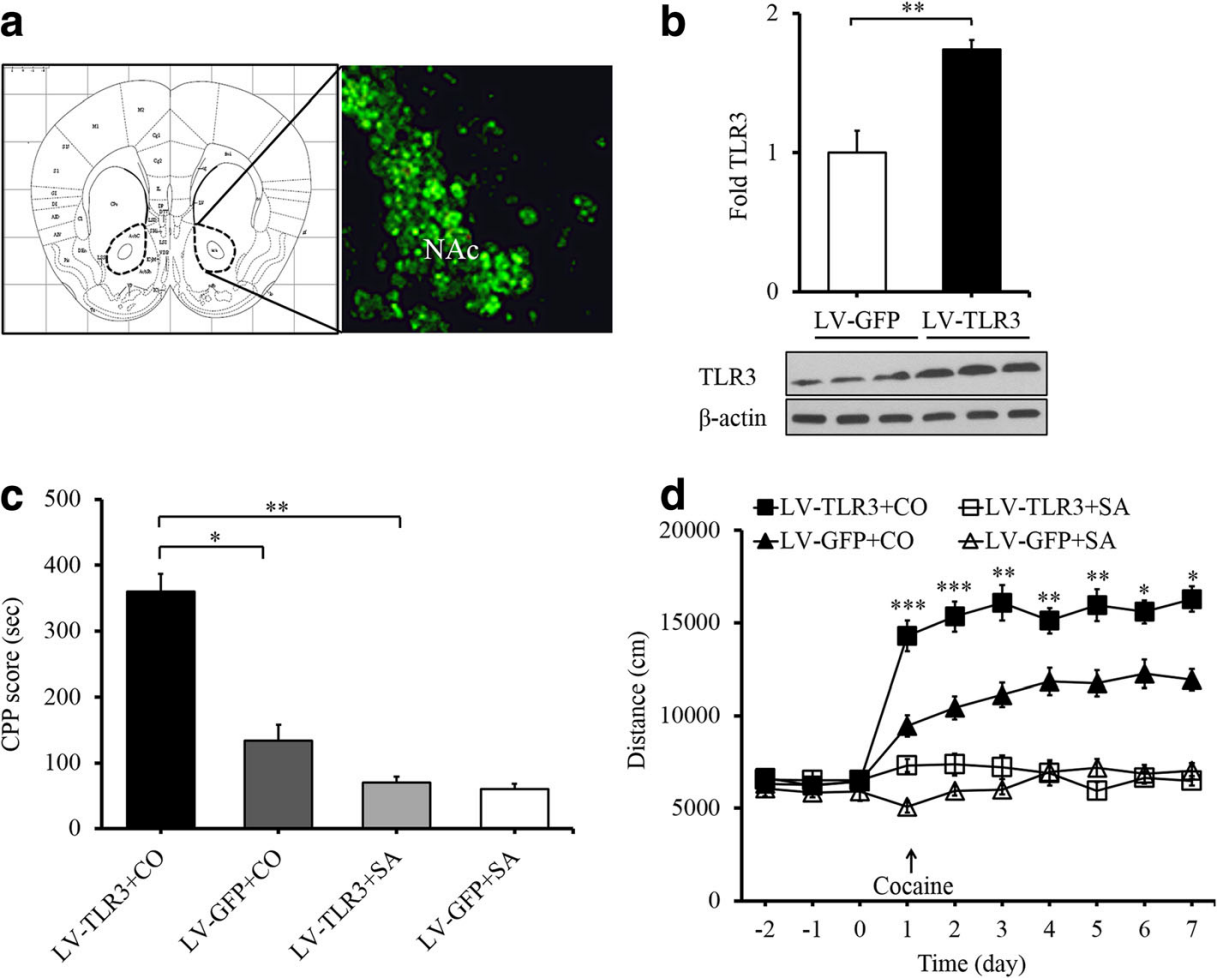
Restoring TLR3 expression in the NAc reverts deficits in cocaine-induced behavior. **a** Lentiviral vector LV-TLR3 expression in the nucleus accumbens. The left of the schematic diagram indicates the injection site of nucleus accumbens. **b** Intra-NAc injection of LV-TLR3 increases TLR3 expression in WT mice. **p < 0.01, *n* = 3/LVGFP group, *n* = 3/LVTLR3 group. (**c**) TLR3 re-expression in the NAc markedly increases the preference for cocaine (20 mg/kg) in comparison to TLR3-knockout mice expressing LV-GFP, *p < 0.05, **p < 0.01, *n* = 10/LVTLR3+CO group, *n* = 10/LVTLR3+SA group, *n* = 10/LVGFP+CO group, *n* = 10/LVGFP+SA group. **d** Group mean total distance traveled in centimeters during the 15 mins after cocaine administration every day (20 mg/kg/day). *p < 0.05, **p < 0.01, ***p < 0.001, *n* = 14/LVTLR3+CO group, *n* = 11/LVTLR3+SA group, *n* = 14/LVGFP+CO group, *n* = 11/LVGFP+SA group. Cocaine (CO), saline (SA). Data are presented as the mean ± SEM

To confirm the role of TLR3 in cocaine behavioral effects, we used both gain-of-function and loss-of-function approaches. The TLR3^−/−^ mice received an intra-NAc injection of lentivirus specifically expressing TLR3 (LV-TLR3; Fig. 4a). Moreover, a lentiviral vector expressing green fluorescent protein (LV-GFP) was used as a control. We evaluated the effects of TLR3 overexpression (LV-TLR3) in the WT control animals by Western blot analysis. The results showed that the expression of TLR3 in LV-TLR3 mice was significantly increased compared to that in LV-GFP mice (Fig. 4b; *t*_(4)_ = 6.558, **p < 0.01). Importantly, we found that the TLR3^−/−^ mice that received an intra-NAc LV-TLR3 injection showed a marked increase in the preference for cocaine (20 mg/kg) in comparison to those receiving an intra-NAc LV-GFP injection (Fig. 4c; *F*_(3,36)_ = 50.066, *p < 0.05). This result showed that restoring TLR3 expression in the NAc reproduced the cocaine CPP effect in TLR3^−/−^ mice. We continued to investigate whether LV-TLR3 could exhibit a similar effect on the locomotor activity of TLR3^−/−^ mice. No significant group differences were observed between these two groups treated with saline. However, when compared to LV-GFP control mice, LV-TLR3-treated mice showed significantly increased locomotor activity after repeated cocaine administration (20 mg/kg/day; Fig. 4d; *F*_(3,42)_ = 42.882, ***p < 0.001). These results indicate that cocaine-induced CPP and locomotor activity revert deficits after restoring TLR3 expression in the NAc, and support the notion that TLR3 may play a stimulative role in cocaine-induced behavior.

### Inhibition of TLR3 attenuates cocaine-induced CPP and locomotor activity in WT mice

**Fig. 5:**
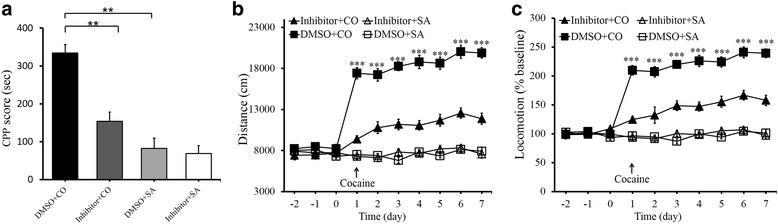
Inhibition of TLR3 weakens cocaine-induced CPP and locomotor activity in wide-type mice. **a** Intra-NAc TLR3/dsRNA complex inhibitor (50 μM, 1 μl/injection) attenuates cocaine CPP (20 mg/kg). **p < 0.01, *n* = 16/DMSO+CO group, *n* = 17/inhibitor+CO group, *n* = 16/DMSO+SA group, *n* = 16/inhibitor+SA group. **b**, **c** Group mean total distance traveled in centimeters in the 15 mins after cocaine administration every day (20 mg/kg/day). DMSO control group showed greater locomotor activity than the inhibitor group over all injection days. ***p < 0.001, *n* = 13/DMSO+CO group, *n* = 13/inhibitor+CO group, *n* = 10/DMSO+SA group, *n* = 11/inhibitor+SA group. Cocaine (CO), saline (SA). Data are presented as the mean ± SEM

TLR3/dsRNA complex inhibitor (#614310, Calbiochem, GER), which is a competitive inhibitor of dsRNA binding to TLR3, was used to inhibit TLR3 [33, 34]. TLR3/dsRNA complex inhibitor was dissolved in DMSO and diluted in pathogen-free PBS before use. The wild-type mice received intra-NAc injections of TLR3/dsRNA complex inhibitor (50 μM, 1 μl/injection), and the control mice received saline. Thirty minutes before CPP measurement, mice were treated with the TLR3 inhibitor or saline. We found that the inhibitor significantly decreased the cocaine CPP (20 mg/kg) in comparison to saline (Fig. 5a; *F*_(3,61)_ = 32.322, **p < 0.01). In addition, the mice injected with the inhibitor exhibited reduced locomotor activity (Fig. 5b; *F*_(3,39)_ = 138.052, ***p < 0.001; Fig. 5c; *F*_(3,39)_ = 100.405, ***p < 0.001). Collectively, TLR3 inhibitor treatment resulted in a significant reduction in cocaine-induced CPP and locomotor activity, and these findings are in line with the effects of TLR3 deficiency.

### TLR3 inhibitor attenuates cocaine-induced upregulation of phospho-NF-κB p65, IKKβ, and p-IκBα

**Fig. 6:**
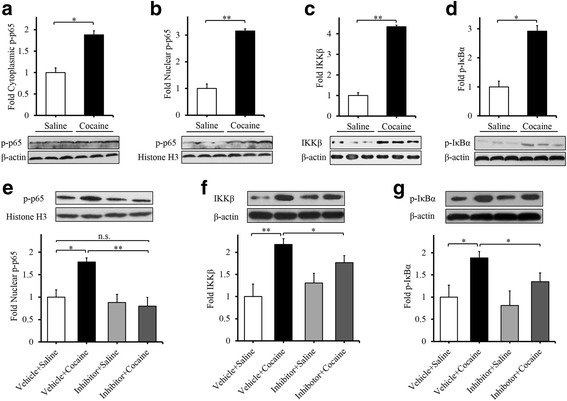
TLR3 inhibitor attenuates cocaine-induced upregulation of NF-κB signaling. Phospho-NF-κB p65 in the cytoplasm (**a**) and nucleus (**b**) in the NAc are significantly increased after cocaine CPP. IKKβ (**c**) and phosphorylated IκBα (**d**) are upregulated. *p < 0.05, **p < 0.01, *n* = 3/saline group, *n* = 3/cocaine group. Intra-NAc Inhibitor (50 μM, 1 μl/injection) decreased cocaine CPP-induced the expressions of nuclear phospho-NF-κB p65 (**e**), cytoplasmic IKKβ (**f**) and phosphorylated IκBα (**g**) in the NAc. *p < 0.05, **p < 0.01, n.s Not significant. *n* = 4/vehicle+saline group, *n* = 4/inhibitor+saline group, *n* = 4/vehicle+cocaine group, *n* = 4/inhibitor+cocaine group. Data are presented as the mean ± SEM

We found that cocaine treatment markedly increased the expression of phospho-NF-κB-p65 both in the cytoplasm and nucleus of cocaine-induced CPP mice (Fig. 6a; *t*_(4)_ = 3.410, *p < 0.05; Fig. 6b; *t*_(4)_ = 5.964, **p < 0.01), which is consistent with previous studies. Interestingly, such effect was clearly attenuated by TLR3 inhibitor when compared to the saline group (Fig. 6e; *F*_(3,12)_ = 8.954, **p < 0.01). Moreover, a significant upregulation of IKK (Fig. 6c; *t*_(4)_ = 7.902, **p < 0.01) and p-IκBα (Fig. 6d; *t*_(4)_ = 2.826, **p < 0.01) was observed in the cytoplasm after cocaine treatment. However, TLR3 inhibitor significantly attenuated cocaine-upregulated expressions of IKKβ and p-IκBα in comparison to the control group (Fig. 6f; *F*_(3,12)_ = 8.703, **p < 0.01; Fig. 6g; *F*_(3,12)_ = 8.124, *p < 0.05). These results suggest that TLR3 modulates cocaine effects possibly through NF-κB signaling.

## Discussion

Previous reports have shown that innate immunity participates in drug-induced behavioral effects through TLRs [9, 35]. In the present study, we investigated the potential role of TLR3 signaling in cocaine addiction. Our results show that inactivation of TLR3 signaling in the NAc, using genetic knockout or pharmacological inhibition, significantly attenuates cocaine-induced CPP, self-administration, and locomotor activity. Our findings show that TLR3 is a novel contributor to cocaine-induced reward behaviors and TLR3/dsRNA complex inhibitor might serve as potential therapeutic targets for the treatment of cocaine addiction.

Increasing evidence show that TLRs, such as TLR4 and TLR2, play a role in drug dependence [5, 35–37]. Long-term ethanol treatment induces an anxiogenic-related behavior in ethanol-deprived WT mice but not in TLR4^−/−^ or TLR2^−/−^ mice [16]. The main symptoms of morphine withdrawal are significantly attenuated in TLR2 knockout mice [38]. Meanwhile, significantly lower expression of cytokines and chemokines were observed in TLR2^−/−^ mice when compared to WT mice after ethanol or morphine withdrawal. Chronic delivery of (+)-naltrexone, a TLR4 antagonist, during the heroin withdrawal phase decreases cue-induced heroin seeking [39]. The previous study showed that cocaine reward and reinforcement require TLR4 signaling [9], TLR4 deficiency alters NAc medium spiny neurons synaptic physiology and drug reward learning [40]. However, a recent study revealed that (+)-naloxone and (+)-naltrexone showed non-specific involvement on behavioral abuse-related effects of cocaine and opioids, as they also decreased food-maintained responding [41]. These studies suggest that cocaine interacts with TLR4 to initiate central innate immune signaling, and pharmacological inhibition of the TLR4 complex suppresses cocaine-induced reinforcing behaviors, but lack of specificity. In our study, when compared to WT mice, cocaine-induced CPP and self-administration were significantly attenuated in TLR3^−/−^ mice. Similarly, TLR3^−/−^ mice exhibited reduced locomotor activity. Pharmacological inhibition of TLR3 also resulted in attenuated CPP score and locomotor activity in cocaine-treated mice. In contrast, intra-NAc injections of lentivirus expressing TLR3 in TLR3^−/−^ mice reversed such effects. Behavioral sensitization shares similar neuroadaptations that underlie relapse to compulsive drug-seeking and taking behavior [42]. These results indicate that TLR3 mediates general aspect of cocaine addiction-related behaviors. One of the future directions is what specific adaptations of TLR3 occur in the NAc in response to cocaine.

How central immune signaling interacts with mesolimbic reward pathways has not been thoroughly elucidated to date. Within the central nervous system, TLRs can identify “molecular patterns” as “non-self” or “danger” signals [43]. They have explicitly evolved to recognize multiple diverse conserved pathogen-associated molecular patterns (PAMPs). Moreover, TLR3 can recognize various and self-RNAs derived from damaged cells. One previous study has reported that TLR3 detects self-noncoding RNA damaged by ultraviolet radiation [44]. U1 RNA, a small nuclear RNA that forms four stem-loops, is released from keratinocytes after ultraviolet B exposure. This self-RNA is detected by TLR3 which then triggers an inflammatory response. Therefore, TLR3 may rely on these loop domains to identify U1 RNA. In addition to U1, other RNAs that are released from damaged tissue could serve as endogenous ligands for TLR3 through their secondary structures [45]. Moreover, cancer therapeutic RNA compounds induce cell death in tumors and serve as TLR3 ligands [46]. Therefore, identification of specific alterations in noncoding RNA induced by cocaine exposure may help to determine the mechanism of TLR3 activation and downstream NF-κB signaling. Long noncoding RNAs (lncRNA) are RNA transcripts longer than 200 nucleotides that do not encode proteins. Various functional lncRNAs possess secondary structures that include multiple regions of dsRNA repeats. They form modular functional domains through these secondary structures that are capable of combinatorially coordinating RNA-RNA, RNA-protein, and RNA-DNA interactions [47, 48]. Our previous study identified numerous lncRNAs that are dysregulated in the NAc of cocaine-conditioned mice [21]. A study also found dysregulated lncRNAs in the midbrain of human cocaine abusers [20]. For example, PRKCQ-AS1, which is the antisense strand of the protein coding transcript PRKCQ, is markedly decreased. It is noteworthy that PRKCQ protein interacts with GCK-like kinase (GLK) to activate the transcription factor NF-κB. Recent studies have reported widespread changes in the expression of lncRNAs during the activation of innate immune responses, and these lncRNAs control important aspects of immunity [49]. Taking into account these findings together with our observations, we speculate that TLR3 may be activated by cocaine-induced lncRNAs. Further studies are needed to elucidate this phenomenon.

However, other possibilities should also be considered. For example, TLR4 has been classically characterized as the innate immune receptor responsible for detecting endotoxins [50], and TLR4 directly binds to the ionotropic glutamate receptor *N*-methyl-d-aspartate (NMDA) subunit 1 (GluN1) in response to lipopolysaccharide [51]. Moreover, cocaine induces changes in NMDA receptors expression in NAc [52]. Interestingly, a recent report showed that cocaine can interact with the TLR4/MD-2 complex [9]. Both in silico and in vitro biophysical studies confirm the relevant physicochemical interactions between cocaine and TLR4/MD2 complex. In addition to cocaine, other addictive substances such as morphine and long-lasting ethanol metabolite can dock to the MD-2 portion of the TLR4 receptor complex in silico and in vitro [53, 54]. Thus, whether or not TLR3 can recognize and interact with cocaine or its metabolites, such as benzoylecgonine and ecgonine methylester, or binds to NMDA receptors requires further investigation [55].

As an important TLR3 downstream transcription factor, NF-κB is best understood in the context of its response to infection and cellular stress [56]. NF-κB has been shown to regulate cocaine-induced synaptic plasticity and addiction [25]. Chronic administration of cocaine increases the levels of two NF-κB subunits, p105/p50 and p65/Rel-A, in the NAc [26]. Meanwhile, the transcriptional activity of NF-κB is elevated throughout the NAc and dorsal striatum in chronic cocaine treated mice. Conversely, inhibition of NF-κB blocks the rewarding effects of cocaine [10]. It has been known that several NF-κB subunits are among the target genes of ΔFosB in the NAc and that cocaine-activated NF-κB is mediated by ΔFosB [26]. However, there are still many other factors in the immune system that can mediate NF-κB activity, such as the TLRs, TNFR family, and IL-1R [57, 58]. In central nervous system, TNF-α/TNFR2-induced neurite regrowth occurs primarily through EphB2 signaling via NF-κB activation [59]. However, one study reported that microglial TNF-α suppresses cocaine-induced neuroplasticity and behavioral sensitization [60]. Astrocytic TLR3 is associated with ischemic preconditioning-induced protection against brain ischemia in rodents via the stimulation of NF-κB [61]. Inhibition of IL-1β signaling attenuates tau pathology and restores neuronal β-catenin pathway function by reducing NF-κB activity in an Alzheimer’s disease model [62]. In our study, we found that cocaine treatment led to the activation of NF-κB through IKK-activated IκBα. Moreover, intra-NAc injection of TLR3 inhibitor decreased the expression of nuclear NF-κB, cytoplasmic IKKβ, and phosphorylated IκBα in the NAc of cocaine-treated mice. These results indicate that TLR3 blockade attenuates the changes of NF-κB signaling in the NAc in response to cocaine, which might underlie the modulatory effect of TLR3 on cocaine effects.

There are still several questions that are worthy of discussion. For example, it is not known whether other downstream factors of TLR3 besides NF-κB, such as IRF-3, IRF-7, and AP-1, are involved in cocaine-induced behavioral effects. Independent TLR3 signaling requires the adaptor molecule TRIF to activate downstream signaling pathways. Ethanol can activate microglia and stimulate NF-κB, AP1, and MyD88-independent IRF3 pathways to trigger the production of inflammatory mediators via TLR4 [63], thus resulting in behavioral impairments [64]. In cultured astrocytes, ethanol treatment activates NF-κB and AP-1 through TLR4 [65]. AP-1 is involved in transcriptional regulation of the human μ-opioid receptor gene [66]. Inhibition of AP-1 activity alters cocaine-induced gene expression and potentiates sensitization [67]. As other downstream molecules of TLRs pathways have been shown to mediate the action of drugs of abuse, the contribution of these factors activated by TLR3 to cocaine effects needs to be investigated further.

## Conclusions

Taken together, the results of this study provide evidence that TLR3 plays a critical role in cocaine-induced behavioral effects and further insight into the link between innate immunity and drug dependence. We propose that TLR3 blockade could be a novel approach to treat cocaine addiction.

## Additional file

Additional file 1: Supplementary material for ORF of LV-TLR3. (DOCX 14 kb)

## Abbreviations

ANOVA: One-way analysis of variance
CPP: Conditioned place preference
GFP: Green fluorescence protein
KO: Knockout
LV-GFP: Lentivirus expressing GFP
LV-TLR3: Lentivirus-mediated re-expression of *Tlr3*
NAc: Nucleus accumbens
NMDA: *N*-Methyl-d-aspartate
PPRs: Pattern-recognition receptors
TLRs: Toll-like receptors
TRIF: TIR-domain-containing adapter-inducing interferon-β
WT: Wild-type
