# Supplementary material for: Toll-like receptor 3 modulates the behavioral effects of cocaine in mice
Source: J Neuroinflammation. 2018 Mar 23;15:93. doi: 10.1186/s12974-018-1130-8 (PMC5865345; doi:10.1186/s12974-018-1130-8)
Supplement: Supplementary file 1 — Supplementary material for ORF of LV-TLR3. (DOCX 14 kb) [file 12974_2018_1130_MOESM1_ESM.docx]

Additional file 1: Supplementary Material for ORF of LV-TLR3

ORF Sequence Information for EX-Mm13690-Lv201 (EX-Tlr3-Lv201)

>EX-Mm01127-Lv201 ORF sequence

atgaaagggtgttcctcttatctaatgtactcctttgggggacttttgtccctatggattcttctggtgtcttccacaaaccaatgcactgtgagatacaacgtagctgactgcagccatttgaagctaacacacatacctgatgatcttccctctaacataacagtgttgaatcttactcacaaccaactcagaagattaccacctaccaactttacaagatacagccaacttgctatcttggatgcaggatttaactccatttcaaaactggagccagaactgtgccaaatactccctttgttgaaagtattgaacctgcaacataatgagctctctcagatttctgatcaaacctttgtcttctgcacgaacctgacagaactcgatctaatgtctaactcaatacacaaaattaaaagcaaccctttcaaaaaccagaagaatctaatcaaattagatttgtctcataatggtttatcatctacaaagttgggaacgggggtccaactggagaacctccaagaactgctcttagcaaaaaataaaatccttgcgttgcgaagtgaagaacttgagtttcttggcaattcttctttacgaaagttggacttgtcatcaaatccacttaaagagttctccccggggtgtttccagacaattggcaagttattcgccctcctcttgaacaacgcccaactgaacccccacctcacagagaagctttgctgggaactttcaaacacaagcatccagaatctctctctggctaacaaccagctgctggccaccagcgagagcactttctctgggctgaagtggacaaatctcacccagctcgatctttcctacaacaacctccatgatgtcggcaacggttccttctcctatctcccaagcctgaggtatctgtctctggagtacaacaatatacagcgtctgtcccctcgctctttttatggactctccaacctgaggtacctgagtttgaagcgagcatttactaagcaaagtgtttcacttgcttcacatcccaacattgacgatttttcctttcaatggttaaaatatttggaatatctcaacatggatgacaataatattccaagtaccaaaagcaataccttcacgggattggtgagtctgaagtacctaagtctttccaaaactttcacaagtttgcaaactttaacaaatgaaacatttgtgtcacttgctcattctcccttgctcactctcaacttaacgaaaaatcacatctcaaaaatagcaaatggtactttctcttggttaggccaactcaggatacttgatctcggccttaatgaaattgaacaaaaactcagcggccaggaatggagaggtctgagaaatatatttgagatctacctatcctataacaaatacctccaactgtctaccagttcctttgcattggtccccagccttcaaagactgatgctcaggagggtggcccttaaaaatgtggatatctccccttcacctttccgccctcttcgtaacttgaccattctggacttaagcaacaacaacatagccaacataaatgaggacttgctggagggtcttgagaatctagaaatcctggattttcagcacaataacttagccaggctctggaaacgcgcaaaccccggtggtcccgttaatttcctgaaggggctgtctcacctccacatcttgaatttagagtccaacggcttagatgaaatcccagtcggggttttcaagaacttattcgaactaaagagcatcaatctaggactgaataacttaaacaaacttgaaccattcatttttgatgaccagacatctctaaggtcactgaacctccagaagaacctcataacatctgttgagaaggatgttttcgggccgccttttcaaaacctgaacagtttagatatgcgcttcaatccgttcgactgcacgtgtgaaagtatttcctggtttgttaactggatcaaccagacccacactaatatctctgagctgtccactcactacctctgtaacactccacatcattattatggcttccccctgaagcttttcgatacatcatcctgtaaagacagcgccccctttgaactcctcttcataatcagcaccagtatgctcctggtttttatacttgtggtactgctcattcacatcgagggctggaggatctctttttactggaatgtttcagtgcatcggattcttggtttcaaggaaatagacacacaggctgagcagtttgaatatacagcctacataattcatgcccataaagacagagactgggtctgggaacatttctccccaatggaagaacaagaccaatctctcaaattttgcctagaagaaagggactttgaagcaggcgtccttggacttgaagcaattgttaatagcatcaaaagaagccgaaaaatcattttcgttatcacacaccatttattaaaagaccctctgtgcagaagattcaaggtacatcacgcagttcagcaagctattgagcaaaatctggattcaattatactgatttttctccagaatattccagattataaactaaaccatgcactctgtttgcgaagaggaatgtttaaatctcattgcatcttgaactggccagttcagaaagaacggataaatgcctttcatcataaattgcaagtagcacttggatctcggaattcagcacattag
